# Estimating the incidence reporting rates of new influenza pandemics at an early stage using travel data from the source country

**DOI:** 10.1017/S0950268813002550

**Published:** 2013-10-10

**Authors:** K. C. CHONG, H. F. FONG, C. Y. ZEE

**Affiliations:** 1 Division of Biostatistics, Jockey Club School of Public Health and Primary Care, The Chinese University of Hong Kong, Hong Kong SAR, China; 2 Center for Global Public Health, University of California, Berkeley, CA, USA

**Keywords:** Influenza, mathematical modelling, surveillance, Susceptible-Infected-Removed (SIR) model, travellers' infection

## Abstract

During the surveillance of influenza pandemics, underreported data are a public health challenge that complicates the understanding of pandemic threats and can undermine mitigation efforts. We propose a method to estimate incidence reporting rates at early stages of new influenza pandemics using 2009 pandemic H1N1 as an example. Routine surveillance data and statistics of travellers arriving from Mexico were used. Our method incorporates changes in reporting rates such as linearly increasing trends due to the enhanced surveillance. From our results, the reporting rate was estimated at 0·46% during early stages of the pandemic in Mexico. We estimated cumulative incidence in the Mexican population to be 0·7% compared to 0·003% reported by officials in Mexico at the end of April. This method could be useful in estimation of actual cases during new influenza pandemics for policy makers to better determine appropriate control measures.

## INTRODUCTION

During the early outbreak of an influenza pandemic, rapid disease transmission can lead to exponential rises of influenza cases throughout the population. Underreporting of influenza cases in early stages poses problems in estimating both pandemic severity and transmission intensity. Underreporting stems from the short infectious periods of influenza infections; thus, individuals may recover before seeking treatment from their healthcare provider or before being tracked in a surveillance system. Asymptomatic or mild cases may not even be reported at all. A previous study has shown that official surveillance only reveals a small proportion of actual infections during influenza pandemics [1] – in some instances the consultation rate in influenza-like illness case-patients was no more than 50%. Furthermore, cases increase exponentially during the initial stage of an outbreak and the limited capacity of surveillance systems, such as limited serological tests, can also lead to underreporting [2].

Underreporting has consequential effects on public health response. From a policy perspective, underreporting can lead to officials underestimating public health risk which in turn affects planning and the implementation of systematic control and prevention activities. For example, there may be a delay in implementing entry screening for travellers or inadequate warning to local and national health departments. The underestimation of incidence and pandemic severity can also reduce education and health notices to the general public about the influenza virus, causing the public not to take measures to protect themselves through vaccines, hand washing, or other control measures. On the other hand, the case-fatality rate would be overestimated as being higher than it actually was due to the missing calculation of asymptomatic and mild cases from the rate denominator [3]. If there is an insufficient system for pandemic control, this situation can place unexpected, unnecessary financial and human resource demands on a healthcare system. Therefore, reliable methods to estimate the reporting rate during early influenza epidemic outbreaks are critical to good public health and infectious disease response systems.

The influenza A(H1N1) pandemic in Mexico in mid-March 2009 is one example of when country officials underestimated influenza incidence rates. Although it was not a peak season for an influenza outbreak, routine influenza surveillance identified an unexpected increase in cases of an influenza-like illness in mid-April 2009 [4]. An acute respiratory illness was discovered in two children and further confirmed as a new strain of H1N1 virus. Subsequently, on 26 April 2009, the World Health Organization (WHO) notified the public of the new H1N1. Additional cases were soon discovered in the USA [5], and the WHO had raised the H1N1 pandemic alert level to phase 5 by the end of April. At the time, governments and the public still lacked sufficient knowledge about the early stages of the outbreak. At this time, according to H1N1 surveillance data from the Ministry of Health in Mexico, cumulative incidence was measured to be as low as 0·003% in Mexico's population [6].

Due to the increasing awareness of H1N1 throughout April 2009, other at-risk countries began control measures at border points of entry to prevent local epidemics. For example, thermal screening was implemented and suspected cases with a travel history to Mexico were monitored and some quarantined [3]. Because surveillance at the borders was quite thorough for influenza-like illness cases even before the H1N1 virus had spread globally, data on early cases such as time of import from the source country is relatively more complete and timely than other available data. For estimating the size and local expansion of the influenza pandemic, this is a valuable data source that also provides a perspective on how the disease is spread.

Previous studies have demonstrated the usefulness of mathematical modelling in summarizing the epidemiology of infectious illness and in examining impact of the diseases from the external factors [7–14]. In this study, a mathematical modelling approach was adopted to develop a method to help quantify the spread of infectious disease in the population. The method is able to estimate the incidence reporting rate by using the local routine surveillance data with estimates refined from statistics of travellers from the source country for an influenza pandemic. The approach made use of the 2009 pandemic influenza A(H1N1) (pH1N1) outbreak as an example.

## METHODS

### Mathematical model

We adopted a susceptible-exposed-infectious-recovered (SEIR) model to describe the dynamic system of the infectious disease [15]. For each time point *t*, a whole population is classified into one of four groups (‘compartments’): susceptible [*S*(*t*)]; exposed [*E*(*t*)]; infectious [*I*(*t*)]; or recovered [*R*(*t*)]. Using *S, E, I*, and *R* to represent each compartment, the SEIR model has four differential equations describing the rates of subject movement for each time step:

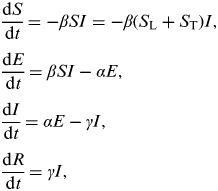
In this compartmental model, once a susceptible individual (including local residents and travellers) in compartment *S*(*t*) is infected, they move to compartment *E*(*t*) and remain there for the latent period. When that latent period is over, they move to compartment *I*(*t*) during the infectious period. When the infectious period is over, individuals in compartment *I*(*t*) recover and move to compartment *R*(*t*). *S*_L_ is the local susceptible size and *S*_T_ is the number of travellers from the source country. As *S*_T_ is far smaller than *S*_L_ i.e. *S*_L_ ≫ *S*_T_, we approximate

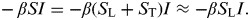


In the model, the probability of an individual becoming infected is configured using the basic reproduction number (*R*_0_), the average number of secondary infections produced by a typical infectious individual in a wholly susceptible population. The transmission rate is *β*, so the force of infection is *βI*. The total population size *N*, is equal to *S* *+* *E* *+* *I* *+* *R* for any time and *N* *=* *S* for time zero. We assumed the lengths of the latent period and the infectious period follow exponential distributions and their averages would be 1/*α* and 1/*γ*, respectively. Adopting the linearization method [16], the basic reproduction number *R*_0_ is equal to *βN/γ*.

### Parameter estimation

In our model, we assumed homogenous mixing between individuals in the system being studied, and that cases reported to officials when infectious. Since the numbers of asymptomatic and non-severe cases may not have been presented for the observed surveillance time-series data *U*(*t*), we used *f*_*t*_(.) to represent a functional form of reporting rates. Therefore, *f*_*t*_(.) is defined as (reported cases/actual cases), *f*_*t*_(.) = *U*(*t*)/*αE*. The *αE* is generated from the SEIR model. We considered two forms of *f*_*t*_(.) in the estimation:

(1) Constant reporting rate: *f*_*t*_(*r*) *=* *r*,
(2) Linearly increasing reporting rate:

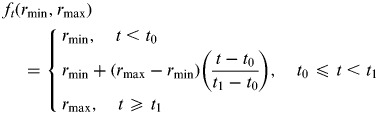
For parameter estimation, we first iterated the parameters by fixing their values within a grid search. Thus the reproduction number *R*_0_ can be fitted into the SEIR model using the least-squares method. We then adopted the earliest times of infected cases imported from Mexico (*T*_*i*_) and the daily rate of travel (*m*_*i*_) to particular country *i*. Assuming the travelling cases had the same daily risk of exposure to the influenza virus as local cases, the average imported cases to a country *i* will be *βm*_*i*_*I*(*t*) for time *t* with *m*_*i*_ the daily rate of travel using the fitted SEIR model. Assuming a Poisson event, we assume the probability of importing at least one case from the source country at time *t* as *p*_*i*,*t*_ =  (1 − *q*_*i*_)(1−exp[−*βm*_*i*_ *I*(*t*)]), where *q*_*i*_ is the entry screening sensitivity for case detection of country *i*. Therefore, the estimated time of the first imported case can be simulated as 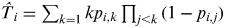. Iterations were repeated for ranges of fixed values within the grid search. Optimum parameters in *f*_*t*_(.) were obtained with the minimum square root of the sum of standardized squared errors (RSE) between observed data (*T*_*i*_) and the simulated estimate times of the first cases imported (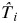) from Mexico, i.e.

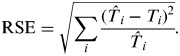
We also developed a bootstrap method to calculate 95% confidence intervals (CIs). Supposing pairs of resample (*T*_i_*, *m*_i_*) were randomly drawn from the original pairs of *T*_*i*_ and *m*_*i*_ with replacement, the bootstrapped RSE for the *j*th iteration of bootstrapping was:

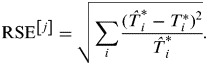
One thousand bootstrapped RSE^[*j*]^ was generated with corresponding fitted parameters. The 2·5th and 97·5th percentiles of the fitted parameters were the lower and upper limits, respectively, of the non-parametric 95% CI over the 1000 samples.

Given the estimate *R*_0_, we back-calculated the exponential growth rate of the pandemic [17]:

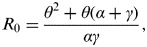
where *θ* is the exponential growth rate. The date of pH1N1 seeding can be calculated as

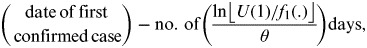
assuming an exponential growth during the early phase of the pandemic.

The estimation method was implemented using SAS v. 9.2.1 software (SAS Institute Inc., USA).

### Materials and parameter values

**Fig. 1.**
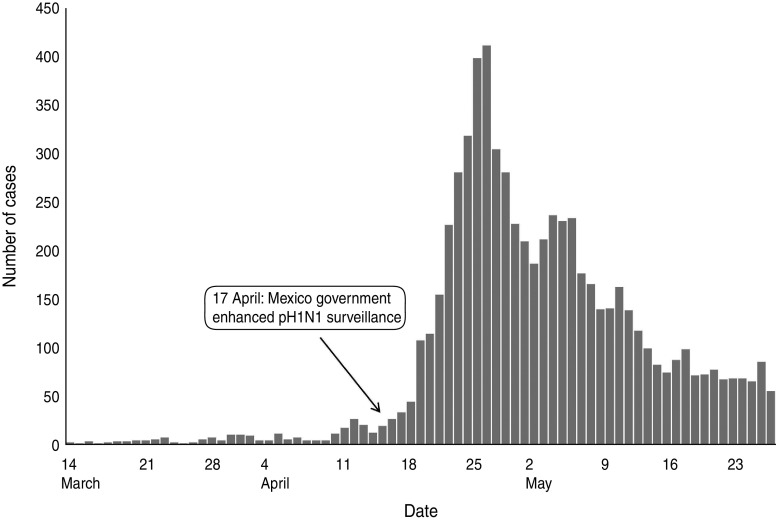
Confirmed cases in Mexico between 14 March 2009 and 27 May 2009.

The population of Mexico (*N*) was 106 682 518 in 2009, a figure provided by the National Council for Population of Mexico [18]. The pH1N1 surveillance data [*U*(*t*)], shown in Figure 1, was obtained from the Ministry of Health of Mexico covering the first wave of the pandemic from 14 March 2009 to 27 May 2009 [6]. We assumed the reporting rate remained constant throughout the time period and increased linearly. In the linear increase approach, the start date (*t*_0_) of enhanced surveillance in Mexico was 17 April 2009 [19] and the end date (*t*_1_) was 17 May 2009.

Traveller data including earliest dates of infected cases arriving from Mexico into different countries are shown in Table 1. We estimated the daily rates of travel to a particular country *i* (*m*_*i*_) by dividing the passenger count in March 2009 and April 2009 by 61 days. We excluded the USA from our study because air travel is not the only means of cross-border transport between the two countries.

**Table 1.** Number of travellers and earliest date of cases imported from Mexico to a particular country in March and April, 2009

Destination country	Travellers (*n*)	Earliest date (2009)	Reference
Canada	101 313	28 April	[28]
Spain	65 724	28 April	[28]
United Kingdom	20 513	28 April	[28]
Costa Rica	16 950	29 April	[28]
Germany	35 772	30 April	[28]
The Netherlands	27 640	30 April	[34]
France	61 960	1 May	[35]
Colombia	24 535	3 May	[36]
El Salvador	15 090	4 May	[37]
Argentina	24 609	7 May	[38]
Brazil	38 749	7 May	[39]
Cuba	42 802	12 May	[40]

The epidemiological details in the parameter estimation were mostly from the previous findings of pH1N1. The lengths of the latent and infectious period S were set at 1·6 days and 1·4 days, respectively [20–24].

### Sensitivity analysis

Limited entry screening at airports at the initial stage of H1N1 could have led to undetected cases from Mexico in the early stages [25–27], especially since Mexico did not implement exit screening. In our model, in order to consider undetected cases, we tested results with a range of entry-screening sensitivities. As exact entry-screening sensitivities would vary for all countries, we varied the screening sensitivities by uniformly choosing from 30% to 100% for each of the countries in every simulation.

We also performed a multivariate sensitivity analysis on the lengths of the latent and infectious periods. The latent period was assumed to follow a gamma distribution with a mean of 1·6 days and a standard deviation of half a day; the infectious period followed a gamma distribution with a mean of 1·4 days and a standard deviation of half a day.

Parameter distributions were drawn from 1000 simulations.

## RESULTS

**Fig. 2.**
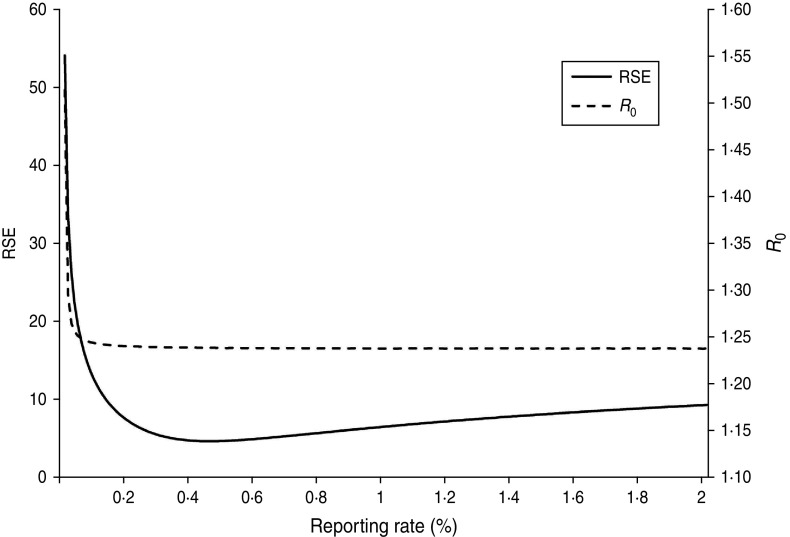
Values of the minimum square root of the sum of standardized squared errors (RSE) and *R*_0_ given different constant *r*.

From our model, the value of the constant reporting rate (*r*) was estimated at 0·46% using a minimum value of RSE. The bootstrapped 95% CI was between 0·28% and 0·69% when the estimated value of *R*_0_ was 1·24 (Fig. 2, Table 2). The value *R*_0_ remained steady when *r* was >0·1%. The figure demonstrated that an increasing reporting rate was associated with exponential decreases in *R*_0_; thus, only fitting the surveillance data to the epidemic model would provide unreliable findings for the estimation of the *r*. Using these estimates, there was a 0·7% (95% CI 0·4–1·1) cumulative incidence in the Mexican population at the end of April 2009, which was the time that the pandemic phase 5 alert level was announced by the WHO.

**Table 2.** Estimates of reporting rates (bootstrapped 95% confidence intervals) given different variations

Variations	Constant	Linear	
	*r* (%)	*r*_min_ (%)	*r*_max_ (%)
None	0·46 (0·28–0·69)	0·46 (0·27–0·68)	0·47 (0·28–0·69)
Entry-screening sensitivity	0·18 (0·09–0·33)	0·10 (0·03–0·26)	0·31 (0·11–0·80)
Lengths of latent and infectious periods	0·44 (0·31–0·69)	0·44 (0·31–0·71)	0·45 (0·32–0·72)

The reporting rate did not increase after enhanced surveillance in Mexico after mid-April 2009, when officials stepped up surveillance systems (Table 2). The *r*_min_ was 0·46% (bootstrapped 95% CI 0·27–0·68), whereas the *r*_max_ was 0·47% (bootstrapped 95% CI 0·28–0·69). Reporting behaviour may not have been significantly affected during this short time-frame.

In the study, we considered the sensitivity of missing ‘detections’ of imported cases in the estimation process. The entry-screening sensitivities of countries were found to be moderately sensitive to our results. If the entry-screening sensitivities were distributed uniformly between 30% and 100%, the constant *r* was estimated as 0·18% (95% CI 0·09–0·31) (Table 2). The value was relatively lower due to a decrease in the average probability of detection. If a linear trend was assumed, a slight increase of the reporting rate was observed. The rate increased from 0·10% (95% CI 0·03–0·26) to 0·31% (95% CI 0·11–0·80). However, this increasing range was insignificant and did not deviate much from our initial estimates.

**Fig. 3.**
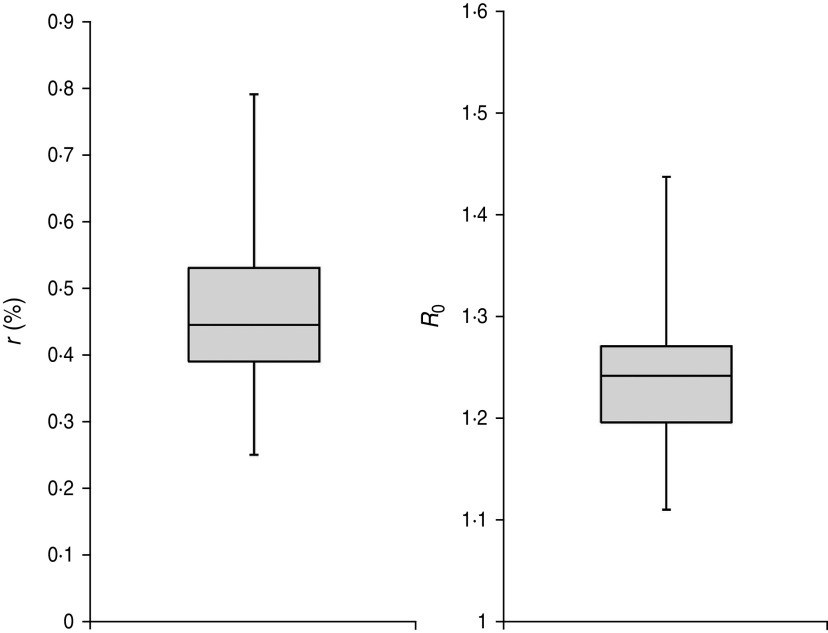
The effect of variations from the length of the latent period (∼gamma[mean = 1·6, s.d. = 0·5]) and the length of the infectious period (∼gamma[mean = 1·4, s.d. = 0·5]) given a constant reporting rate assumption. Left panel is the box-plot of *r* and the right panel is the box-plot of *R*_0_.

**Fig. 4.**
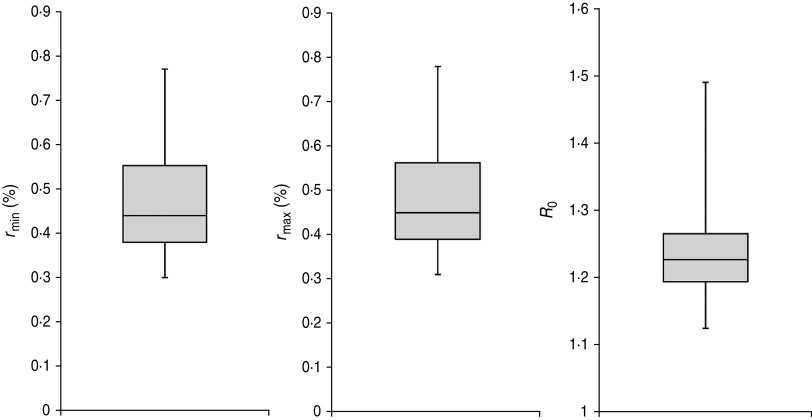
The effect of variations in the lengths of the latent and infectious periods given a linearly increasing reporting rate assumption. The impacts to *r*_min_, *r*_max_, and *R*_0_ are shown by the box-plots from left, middle, and right panels, respectively.

The impacts of variation of latent period length and infectious period on our results were also tested. As shown in Figures 3 and 4, the variation of lengths did not make any impact on the reporting rate estimation. The constant *r* was 0·44% (bootstrapped 95% CI 0·31–0·69) and the values of the linear reporting rates (*r*_min_ and *r*_max_) were both close to this value (Table 2). However, the variations did affect the estimated value of *R*_0_; the estimated median *R*_0_ was 1·24 (range 1·11–1·44) given a constant assumption of *r*. Insignificant difference was observed for the range of *R*_0_ given a linearly increasing assumption. The range of *R*_0_ was consistent with other studies [20, 28, 29].

Given the estimates, the date of seeding for pH1N1 was exponentially interpolated from the date of the first confirmed case (i.e. 14 March 2009 with an estimate of about 400 infections). From the results, the date of seeding for pH1N1 was estimated as 24 December 2011 (95% CI 17–29 December 2011) in order to maintain a sufficient large epidemic size for exportation of cases. About 5500 Mexicans were infected by pH1N1 virus before the date of the first confirmed case in the surveillance data. When the minimum and maximum values (1·11 and 1·44, tespectively) of the range of *R*_0_ were adopted in the estimation, the dates of seeding for pH1N1 were 26 September 2011 (95% CI 12 September 2011 to 7 October 2011) and 27 January 2012 (95% CI 23–30 January 2012), respectively.

## DISCUSSION

A reliable method to estimate reporting rates during early phases of a new influenza pandemic is critical in addressing infectious disease response in the 21st century, especially with increased travel by air, land, and sea [25]. The importance of this was highlighted in Mexico's 2009 influenza pandemic, in which the reported incidence by Mexican officials (0·003%) during the early stages of the outbreak was not even close to our estimate during the early outbreak. Even though the strain of pH1N1 virus had been further confirmed in mid-April 2009, the Mexican officials' reporting rates still did not increase. This situation masked the actual growth of pH1N1, leading to a reduction of public awareness and potentially more rapid disease transmission. Inaccurate estimates overstating the risk can provide misleading information to the public and potentially raise levels of anxiety or panic [30].

A reliable estimate can assist officials at local, national, and global levels in planning and implementing prevention and control strategies for a pandemic influenza during the early stages, and better inform policy and protocols for other infectious disease outbreaks. In our study, we introduced such a method using existing information available to countries during a pandemic, the time at which imported cases may be arriving from a source country, to estimate reporting rates. According to our results, the estimated epidemic size was larger than officially reported in 2009; we found an estimate of 0·7% cumulative incidence (about 691 000 individuals) in the Mexican population compared to the 0·003% reported from the Ministry of Health of Mexico [6]. In terms of the epidemic size, our estimates were in line with other studies [2, 31] but were higher than that of Fraser *et al.* [28]. The reason for the difference is that our approach adopted time-series data for reported incidence, which can help better validate results when using traveller data. Several studies have employed a cross-sectional set of travel data to estimate actual epidemic size but those approaches did not aim to project the epidemic curve or address trends of reporting behaviour.

Interpolated estimation suggested the date of initiation for pH1N1 was late December 2008, which agrees well with other studies [28] and, suggests that the pH1N1 virus had the potential to spread to other continents prior to laboratory confirmation of the virus [29]. With the use of the SEIR model and the estimates, we were also able to estimate that around 0·005% of the Mexican population was infected prior to the first case being detected by the surveillance system. Therefore, there is a possibility that undetected cases from Mexico, in other countries before the first global case was reported, could have affected our estimates. By using the mathematical model, the probability of having imported case from Mexico for at least one listed country (Table 1) was about 0·21 (results not showed) prior to 14 March 2009. Hence, the early ‘missing’ detection of imported cases from Mexico was not unexpected. This situation has similar potential issues with entry-screening sensitivities mentioned previously in the Results section, and we believe it would have only a minor effect on our findings.

The reliability of our proposed method would depend greatly on the quantity and quality of travel surveillance available at the borders during the early stages of a potential pandemic. If surveillance data from travellers could be collected in a timely way, it could effectively align with the estimation of a new influenza pandemic size and threat. However, there are challenges in acquiring large samples because countries especially those which do not border each other have different and incompatible surveillance systems as well as disparate policies on international reporting and collaboration. In our study, we only found 12 countries that reported their confirmed cases with known travel history in Mexico. Regarding this issue of small sample size, a bootstrap method was the preferred choice. In the future, improved coordination and technical innovations to streamline or even centralize infectious disease surveillance of travellers between countries would be beneficial to public health.

Besides the surveillance data from travellers, routine serological surveys could be another source of estimating incidence. However, compared to the surveillance data at borders, routine samples of seroprevalence may not be suitable during an initial outbreak of a pandemic as it requires laboratory resources and a longer collection time [32]. Its reliability also relies on the sampling frame of the data [33]. Using serial cross-sectional serological data along with surveillance data could be reliable in estimating infection rates, since serological data could refine parameter estimates [33]. In order to account for possible estimation errors, multi-faceted surveillance measures are recommended, especially for new outbreaks of influenza pandemics during the early stages.

One of the advantages of using our method is its flexibility in adapting to/incorporating other epidemic models. It can be extended using similar concepts which adapt the reporting rate function of incidence in the epidemic models. For example, our approach could potentially be extended to demographic stratified models. As younger age groups were likely to be affected by pH1N1 and to be presented in ascertainment, incorporation of demographic stratified models would make the modelling results more realistic. However, sufficient data is required to support the extension of the method.

One of the caveats for applying the method to pH1N1 in Mexico was the homogenous dispersion of infections throughout the source country [31]. Clearly, the pH1N1 outbreak may have not yet spread to all cities in Mexico at the early stage. Without available infection data at the city level, the resolution of our results would not be high enough and the spatial variation would alter our estimates. Although our method provides further understanding on how to tackle estimates of incidence reporting rates at early stages of an influenza outbreak, future studies could explore further model extensions.
